# Integrated Analysis Revealing the Senescence-Mediated Immune Heterogeneity of HCC and Construction of a Prognostic Model Based on Senescence-Related Non-Coding RNA Network

**DOI:** 10.3389/fonc.2022.912537

**Published:** 2022-06-30

**Authors:** Yanan Jiang, Kunpeng Luo, Jincheng Xu, Xiuyun Shen, Yang Gao, Wenqi Fu, Xuesong Zhang, Hongguang Wang, Bing Liu

**Affiliations:** ^1^ Department of Pharmacology (State-Province Key Laboratories of Biomedicine- Pharmaceutics of China, Key Laboratory of Cardiovascular Research, Ministry of Education), College of Pharmacy, Harbin Medical University, Harbin, China; ^2^ Translational Medicine Research and Cooperation Center of Northern China, Heilongjiang Academy of Medical Sciences, Harbin, China; ^3^ Department of Gastroenterology and Hepatology, The Second Affiliated Hospital of Harbin Medical University, Harbin, China; ^4^ Department of Oral and Maxillofacial Surgery, The First Affiliated Hospital of Harbin Medical University, Harbin, China; ^5^ School of Civil Engineering, Northeast Forestry University, Harbin, China

**Keywords:** hepatocellular carcinoma (HCC), senescence, non-coding RNA (ncRNA), prognosis, regulatory network

## Abstract

**Background:**

Hepatocellular carcinoma (HCC) is the second leading cause of cancer-related mortality worldwide. Non-coding RNAs play an important role in HCC. This study aims to identify a senescence-related non-coding RNA network-based prognostic model for individualized therapies for HCC.

**Methods:**

HCC subtypes with senescence status were identified on the basis of the senescence-related genes. Immune status of the subtypes was analyzed by CIBERSORT and ESTIMATE algorithm. The differentially expressed mRNAs, microRNAs (miRNAs), and long non-coding RNAs (lncRNAs) were identified between the two HCC subtypes. A senescence-based competing endogenous RNA (ceRNA) co-expression network in HCC was constructed. On the basis of the ceRNA network, Lasso Cox regression was used to construct the senescence-related prognostic model (S score). The prognosis potential of the S score was evaluated in the training dataset and four external validation datasets. Finally, the potential of the prognostic model in predicting immune features and response to immunotherapy was evaluated.

**Results:**

The HCC samples were classified into senescence active and inactivate subtypes. The senescence active group showed an immune suppressive microenvironment compared to the senescence inactive group. A total of 2,902 mRNAs, 19 miRNAs, and 308 lncRNAs were identified between the two subtypes. A ceRNA network was constructed using these differentially expressed genes. On the basis of the ceRNA network, S score was constructed to predict the prognosis of patients with HCC. The S score was correlated with immune features and can predict response to immunotherapy of cancer.

**Conclusion:**

The present study analyzed the biological heterogeneity across senescence-related subtypes and constructed a senescence-related ceRNA-network-based prognostic model for predicting prognosis and immunotherapy responsiveness.

## Introduction

Liver cancer is a major health problem worldwide, accounting for 4.3% of new cancer cases (905,677 cases) and 8.3% of cancer deaths (830,180 deaths) in 2020 globally (1). Hepatocellular carcinoma (HCC) is the commonest type of liver cancer, comprising 75%–85% of cancer cases. Despite the advances in HCC therapy, the 5-year survival rate of advanced HCC is still extremely low and the recurrence rate is relatively high (2, 3). The immune system plays important role in HCC. An immunosuppressive tumor microenvironment would impair the recognition of HCC cells (4). Immunotherapeutic regimens improved the clinical management of cancer. However, there are still many cases that are not response to immunotherapy (5, 6). Due to the heterogeneity of cancer, one of the major challenges in immunotherapy is the identification of accurate biomarkers in predicting responses to immunotherapy.

Aging and cellular senescence is the deterioration of tissues and cells. The elimination of senescent cells could delay aging and protect against aging-related diseases, such as cancer. Aging is also a risk factor of tumorigenesis. The senescence of stromal cells establishes an immunosuppressive microenvironment that facilitated the initiation of tumor (7). The hallmarks of cancer constitute the complexities of neoplastic diseases. Recently, Douglas Hanahan purposed that senescent cells should be considered as a hallmark of cancer (8). A series of senescence markers or features has been identified (9–11). The senescence-related genes were correlated with immune cell (IC) infiltration in the tumor microenvironment and the prognosis of patients with cancer (12). The resistance of HCC to current therapies was mainly associated with the immune microenvironment heterogeneity (13). Senescence could modify the tumor microenvironment and thus impact the response of tumor to immune system and immunotherapies (14). Therefore, senescence activity may be a potential index for predicting the immunotherapy responsiveness.

Non-coding RNAs play an important role in cancer. Plenty of differentially expressed non-coding RNAs are identified by high-through put techniques in HCC tissues (15, 16). Increasing studies revealed the role of non-coding RNAs in HCC. On the basis of competing endogenous RNA (ceRNA) mechanism, the regulatory network exists among mRNAs, microRNAs (miRNAs), and long non-coding RNAs (lncRNAs) (17). For example, MCM3AP-AS1 is an oncogenic lncRNA, which is highly expressed in HCC and positively correlated with poor prognosis of patients with HCC. The knockdown of MCM3AP-AS1 inhibited the progression of HCC by regulating miR-194-5p/FOXA1 axis (18). Similarly, the enhanced expression of lncRNA-PDPK2P was observed in HCC tissues, which is positively correlated with PDPK2P and PDK1. In addition, the expression of lncRNA-PDPK2P was negatively correlated with prognosis of patients with HCC. lncRNA-PDPK2P can promote HCC progression through the PDK1/AKT/Caspase-3 pathway (19). Recently, non-coding RNAs were proved to be involved in the progression of HCC through modulating the process of senescence. Miat was identified as a senescence-associated lncRNA in HCC. LncRNA Miat could increase the expression of Sirt1 by inhibiting miR-22-3p expression. Whereas, knockdown of lncRNA Miat could inhibit HCC progression by promoting cellular senescence (20). In addition, the ceRNA network also associated with the prognosis of cancer (21–23).

The development of high-throughput detection techniques and big data resources provides a method for the explanation of the heterogeneous of cancer and promotes the precise medicine of cancer. In the present study, we identified two subtypes of HCC based on the aging characteristics of tumors. Then, we acquired the differentially expressed mRNAs, lncRNAs, and miRNAs between subtypes and constructed the senescence-mediated HCC regulatory ceRNA network. On the basis of this network, a machine learning–based method was used to construct a prognostic model for HCC. Our results showed that the constructed prognostic model can effectively predict the prognosis of patients with HCC, characteristics of immune microenvironment, and response to immunotherapy.

## Material and Methods

### Data Acquisition and Processing

For the senescence subtype identification, ceRNA network construction, and prognostic model construction, clinic information and transcriptome data of patients were obtained from TCGA-LIHC cohort that is from The Cancer Genome Atlas (TCGA) (24). Data were acquired based on the R package “TCGAbiolinks” (25). To validate the prognostic model’s efficiency, patients’ clinic information and transcriptome data of validation cohort were obtained from data series from the Gene Expression Omnibus (GSE10143, GSE14520, and GSE76427) and data series LIRI-JP, which is from International Cancer Genome Consortium (https://dcc.icgc.org) (26). To explore the potential of our constructed model in predicting response to immunotherapy of cancer patients, immunotherapy cohort “IMvigor210” was enrolled in the study. The detailed information of cohorts was obtained based on the R package “IMvigor210CoreBiologies” (27). In IMvigor210 cohort, PD-L1 expression on ICs is assessed by SP142 immunohistochemistry assay, and the samples were divided into IC0 (<1%), IC1 (≥1% and <5%), and IC2+ (≥5%). Samples of IMvigor210 cohort have also been classified into three immune microenvironment-related subtypes: immune-deserted, immune-inflamed, and immune-excluded. The immune microenvironment characteristics of the three subtypes are as follows. Immune-inflamed subtype is characterized by the presence of various ICs in the tumor parenchyma, and the ICs are positioned in proximity to the tumor cells. Immune-excluded subtype is characterized by the presence of various ICs, whereas the ICs cannot penetrate the tumor parenchyma. Immune-deserted subtype is characterized by the paucity of T cells. Clinic data in this research are all from publicly available data. Thus, local research ethics committee approval is not applicable.

### Identification of Senescence Active and Inactive HCC Subtypes

To identify the senescence active subtype and senescence inactive subtype of HCC, 39 laboratory-validated cancer-associated senescence signatures were acquired from CellAge database (28). Detail information of the signatures is presented in **Supplementary Table 1**. On the basis of the 39 signatures, we conducted hierarchical clustering analysis to identify the senescence active subtype and senescence inactive subtype of HCC. GSVA was used to analyze the senescence activity of the HCC subtypes and further validate the robustness of the HCC subtype identification (29).

### Immune Characteristics Analysis

In this research, CIBERSORT, the deconvolution algorithm, was applied for the calculation of the cell composition of complex tissues to analyze the IC infiltration level in the tumor microenvironment (30). To evaluate the immune activity of cancer, ESTIMATE algorithm was used to calculate the immune score and stromal score of cancer (31).

### Acquisition and Functions Analysis of Differential Expression Genes

Differential expressed mRNAs, miRNAs, and lncRNAs were acquired in the threshold of P < 0.05. Processes were conducted by the R package “edgeR” (32). To investigate the biological functions mediated by the differential expression transcriptomes, we conducted the enrichment analysis based on GO database (33) and KEGG database (34). Analysis was conducted and visualized by Metascape (https://metascape.org/).

### Construction of the Senescence-Based ceRNA Co-Expression Network in HCC

To explore the potential ceRNA-mediated regulatory role of senescence and identify the potential functional non-coding regulatory region, we constructed the senescence-based ceRNA network of HCC. The correlation between senescence signatures and differentially expressed genes (miRNAs, lncRNAs, and mRNAs) was assessed by Spearman correlation coefficients. Correlations with P < 0.05 and R < 0.2 or R > 0.2 were considered significant and submitted to the network construction. The ceRNA network was constructed by assembling all correlation and visualized by Cytoscape software (35).

### Construction of the Senescence ceRNA Network-Based Gene Prognostic Model

First, all senescence-related mRNAs were acquired from the ceRNA network. Univariate Cox regression was applied to identify the one that is correlated with prognosis of patients and submitted to the model construction. Then, the Lasso Cox regression was employed to select the model component genes and calculate their coefficients for the construction of the prognostic model. Finally, the senescence feature-based risk score (S score) was constructed through the integration of mRNA expression level and its coefficients. The detailed information of the selected genes and their coefficients is presented in **Supplementary Table 2**.

### Statistical Analysis

In this research, Wilcoxon rank sum test was used to compare the continuous variables between the two groups. Log-rank test was applied to compare the prognosis of two groups. TimeROC analysis was performed to evaluate prediction efficiency of the S score. The distribution of samples in the two groups was compared by chi-square test. Spearman correlation was used to analyze the correlation of two continuous variables. If not mentioned, P < 0.05 was considered as statistically significant.

## Results

### Identification of the Senescence Active and Inactive HCC Subtypes

**Figure 1:**
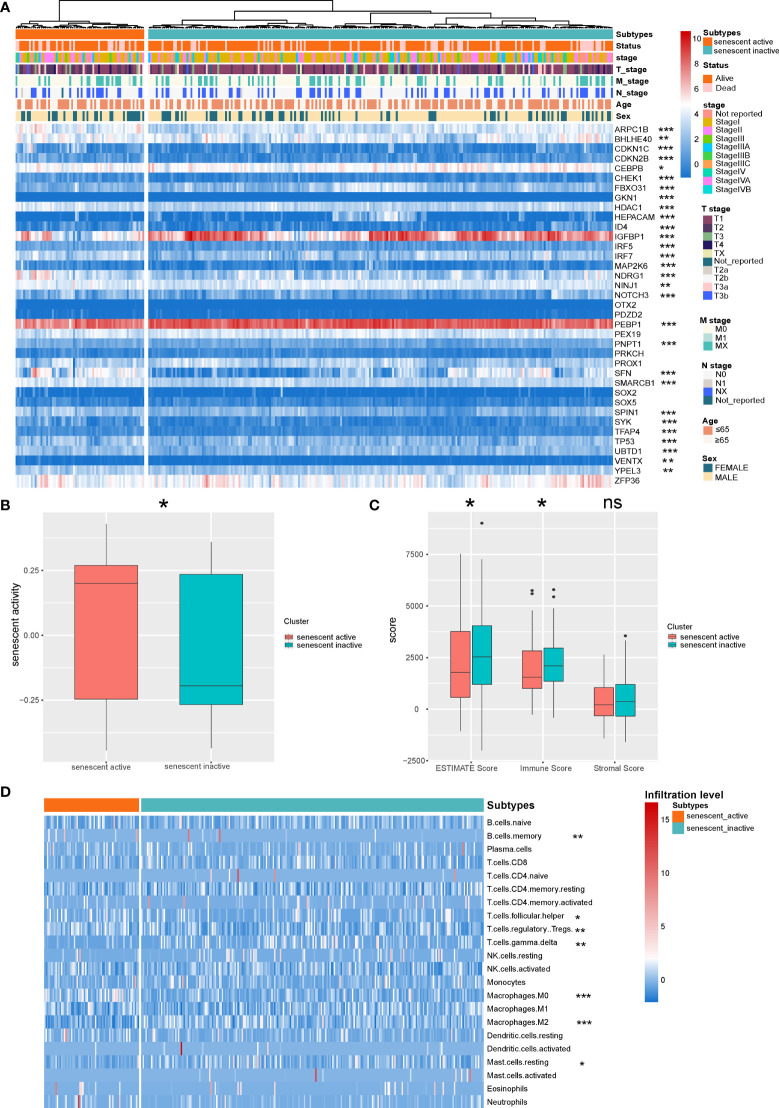
Identification of the senescence-related HCC subtypes. **(A)** Landscape of the senescence-related HCC subtypes. **(B)** Comparison of the senescence activity of the two HCC subtypes. **(C)** Comparison of the ESTIMATE Score of the HCC subtypes. **(D)** Landscape of the immune infiltration level of the two subtypes. *P < 0.05, **P < 0.01, and ***P < 0.001. NS: Not Significant.

Recent research demonstrated that senescence plays an important role during cancer progression (36). We first identified the senescence active and inactive HCC subtypes based on the senescence signatures (**Figure 1A**). To validate the accuracy of the subtype identification, we evaluated the senescence activity of the subtypes by GSVA. According to the result, one subtype has shown significantly higher senescence activity, thus named as senescence active subtype, another subtype named as senescence inactive subtype (**Figure 1B**, P < 0.05). Recent research indicated that senescence is the emerging regulator of the tumor microenvironment alteration (37). Here, we further analyzed the differences of immune features in the two subtypes. ESTIMATE analysis demonstrated that senescence active subtype had the significant lower immune response activity (**Figure 1C**, Immune Score: P < 0.05; ESTIMATE Score: P < 0.05). Next, we then calculated the immune infiltration level of the two subtypes. Two subtypes showed the different immune infiltration features (**Figure 1D**).

### Acquisition of Differential Expressed Genes Between the Two HCC Subtypes

**Figure 2:**
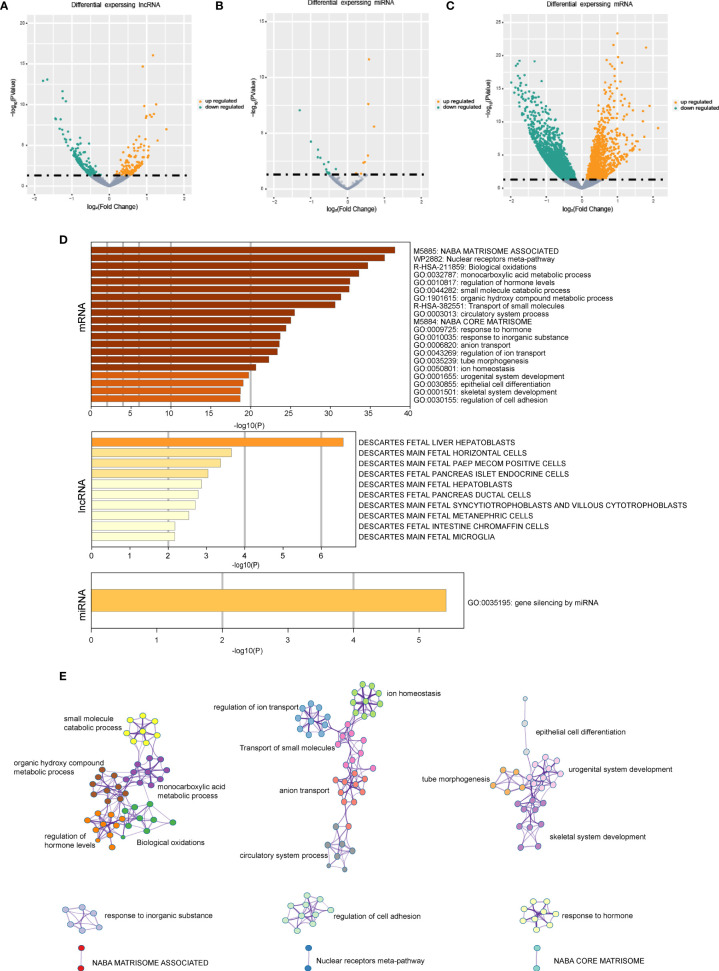
The gene expression difference in the senescence-related HCC subtypes. **(A)** The volcano plots of differential expressed lncRNAs in the two HCC subtypes. **(B)** The volcano plots of differential expressed miRNAs in the two HCC subtypes. **(C)** The volcano plots of differential expressed mRNAs in the two HCC subtypes. **(D)** Enrichment analysis based on differentially expressed genes. **(E)** Network of the differential expressed mRNAs’ significantly enriched pathways.

The differentially expressed mRNAs, miRNAs, and lncRNAs were identified between the two HCC subtypes, respectively. As shown in the volcano plot, a total of 2,902 mRNAs, 19 miRNAs, and 308 lncRNAs were identified (**Figures 2A–C**). Enrichment analysis was employed to investigate the biology functions mediated by the identified genes (**Figures 2D, E**). Top three enrichment terms of the mRNAs were “NABA matrisome associated”, “Nuclear receptors meta-pathway”, and “Biological oxidations”.

### Construction of the Senescence-Related ceRNA Network-Based Prognostic Model

**Figure 3:**
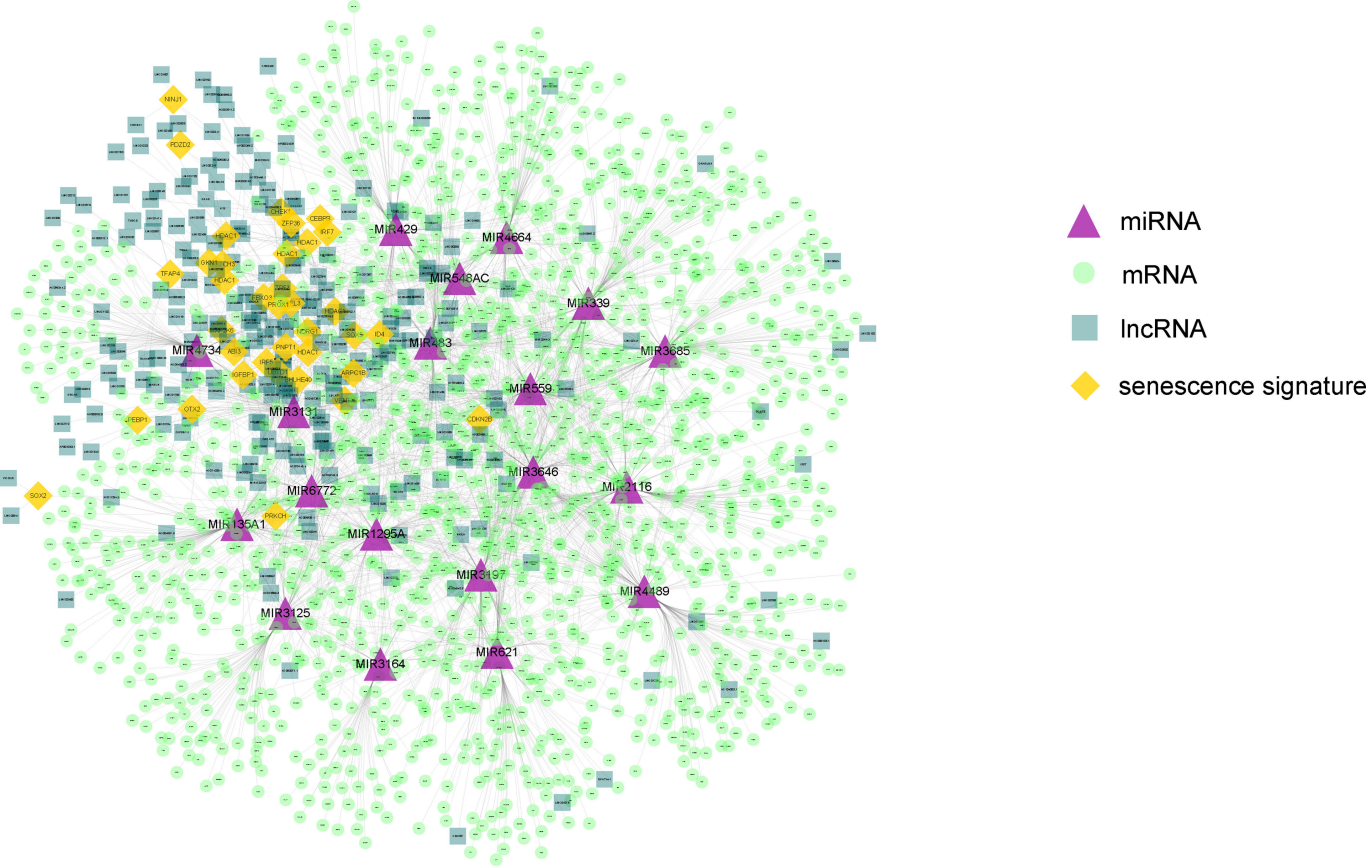
The constructed ceRNA network using differentially expressed genes between the two HCC subtypes. The network contained 39 senescence signatures, 235 lncRNAs, 19 miRNAs, and 1,546 mRNAs regulated by non-coding RNAs. The purple triangle represents miRNAs. The yellow diamond represents senescence signatures. The light green circle represents mRNAs regulated by non-coding RNAs. The dark green square represents differential expressed lncRNAs. The orange diamond represents senescence signatures.

Next, on the basis of the differentially expressed mRNAs, miRNAs, and lncRNAs, we constructed the senescence-mediated ceRNA network. The network contained 39 senescence signatures, 235 lncRNAs, 19 miRNAs, and 1,546 mRNAs regulated by non-coding RNAs (**Figure 3**). The 1,546 mRNAs were submitted for the subsequent construction of prognostic model, which is based on the senescence-related ceRNA network.

**Figure 4:**
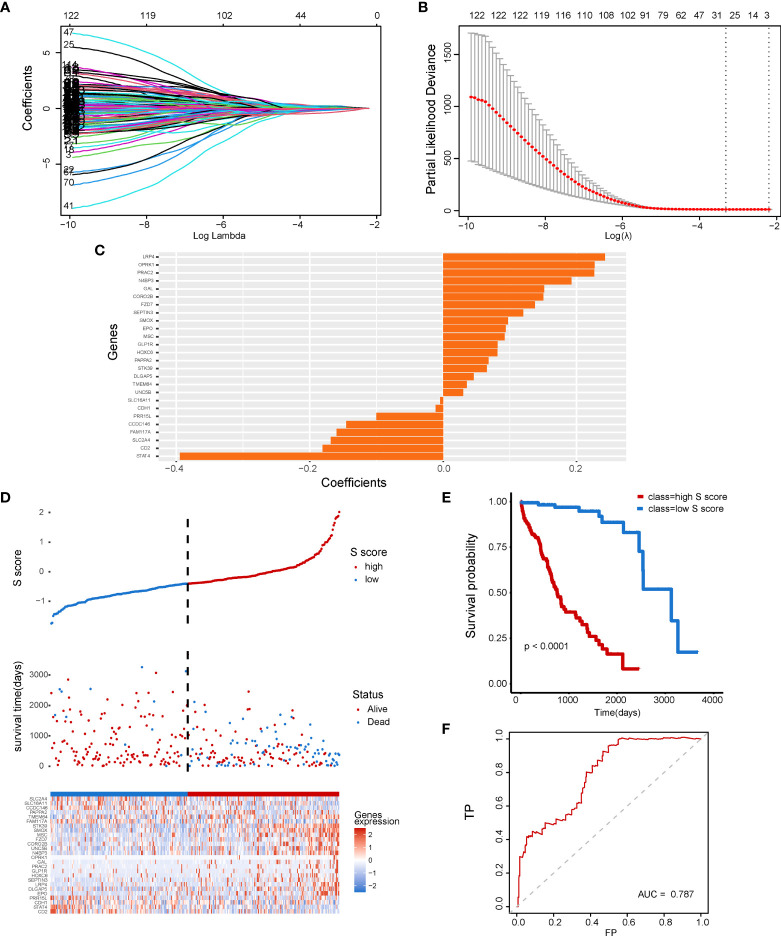
Construction of a senescence-related prognostic model for HCC. **(A)** The coefficients of genes calculated by multivariate Cox regression using LASSO. **(B)** The partial likelihood deviance of genes. **(C)** The coefficients of selected genes. **(D)** S score distribution for patients in the TCGA-LIHC database. **(E, F)** Comparison of patient’s prognosis in high–S score group and low–S score group.

To further explore the clinical application potential of the ceRNA network, we constructed the ceRNA network-based prognostic model (S score) by a machine learning–based method. First, univariate Cox regression was conducted to identify the prognosis related genes from the 1,546 identified mRNAs regulated by non-coding RNAs. A total of 122 genes were selected and used for the Lasso Cox regression to construct the S score (**Figures 4A, B**). The selected genes and their coefficients were presented in **Figure 4C**. To validate the robustness of the S score, the S score of the training cohort was calculated (**Figure 4D**). Our results indicated that the S score can well predict the outcome of patients (**Figure 4E**, P < 0.0001; **Figure 4F**, AUC = 0.787).

### Validation of the Prognostic Efficiency of S Score in External Datasets

**Figure 5:**
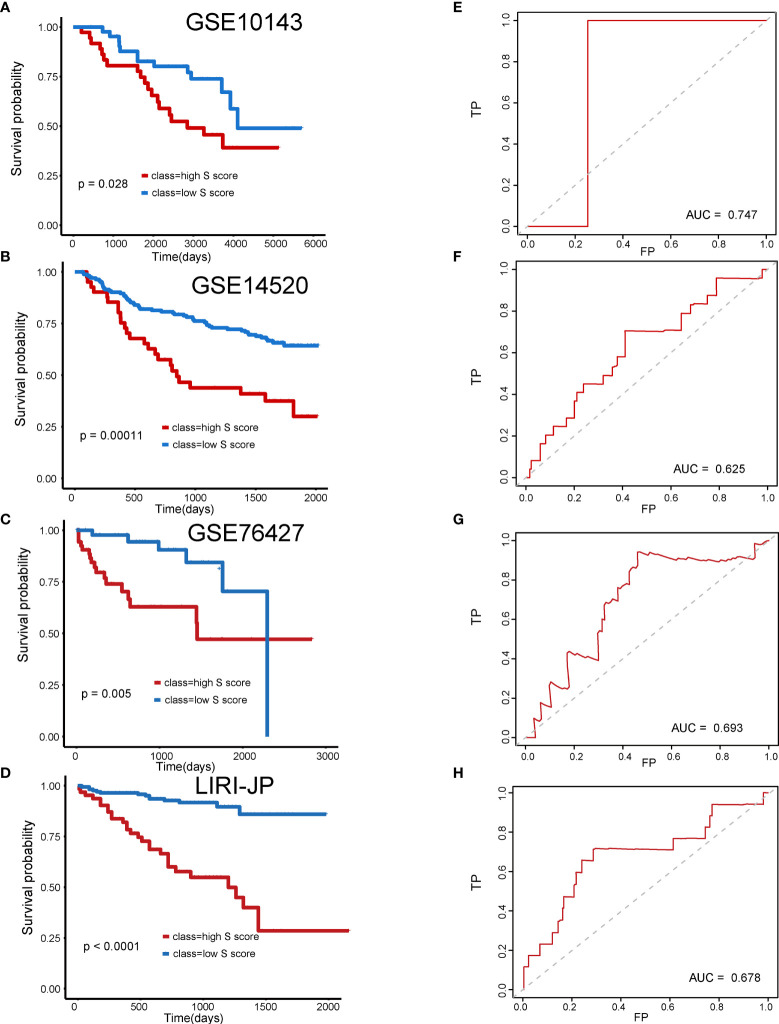
Validation of the prognostic efficiency of S score in external datasets. **(A)** Overall survival of patients in GSE10143. **(B)** Overall survival of patients in GSE14520. **(C)** Overall survival of patients in GSE76427. **(D)** Overall survival of patients in LIRI-JP. **(E)** AUC curve of the patients with HCC in the GSE10143. **(F)** AUC curve of the patients with HCC in the GSE14520. **(G)** AUC curve of the patients with HCC in the GSE76427. **(H)** AUC curve of the patients with HCC in the LIRI-JP.

Subsequently, four external validation datasets, GSE10143, GSE14520, GSE76427, and LIRI-JP, were employed to further validate the prognostic efficiency of the S score. Patients in each dataset were divided into high–S score group and low–S score group, respectively. In datasets GSE10143, GSE14520, GSE76427, and LIRI-JP, patients of the high–S score group all performed worse prognosis than the low–S score group (**Figures 5A, B**: P = 0.028, AUC = 0.747; **Figures 5C, D**: P = 0.00011, AUC = 0.625; **Figures 5E, F**: P = 0.005, AUC = 0.693; **Figures 5G, H**: P < 0.0001, AUC = 0.678). The detailed information of sample distribution is presented in **Supplementary Figure 1**. The integrated results confirmed that the S score has great efficiency in predicting the outcome of patients with HCC.

### The Correlation of the S Score and Immune Features in HCC

**Figure 6:**
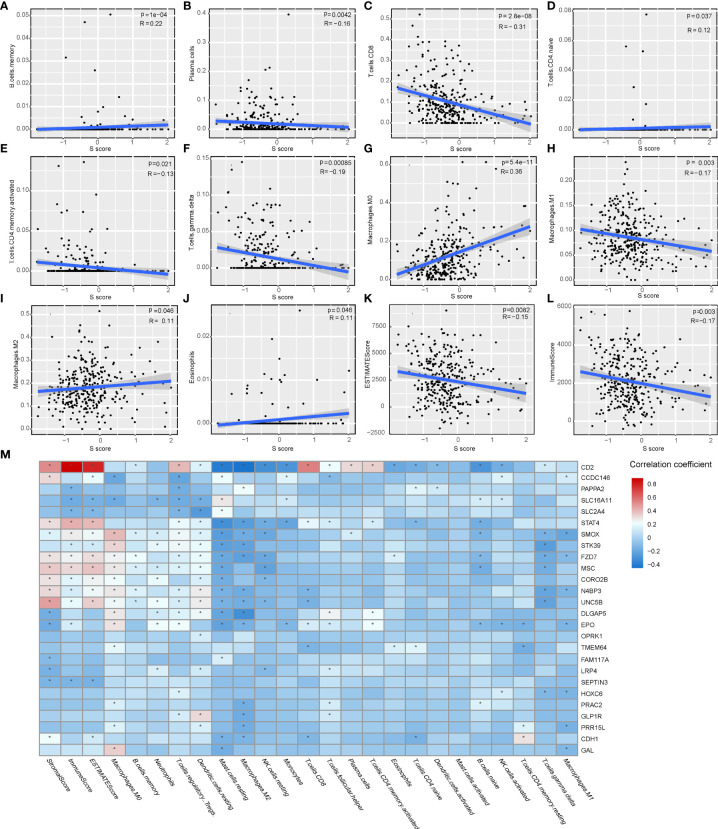
The correlation of the S score and immune features in HCC. **(A–J)** The correlation of S score and IC, including memory B cells **(A)**, plasma cells **(B)**, CD8 T cells **(C)**, naive CD4 T cells **(D)**, activated memory CD4 T cells **(E)**, gamma delta T cells **(F)**, M0 Macrophages **(G)**, M1 Macrophages **(H)**, M2 Macrophages **(I)**, and Eosinophils **(J)**. **(K–L)** The correlation of S score and immune scores, including Immune Score **(K)** and ESTIMATE Score **(L)**. **(M)** Detailed information of the correlation between the S score component genes and immune features. The color bar represents correlation coefficients. *P < 0.05.

The immune features of senescence active and inactive subtype also indicated that senescence mediated an immune-suppressive characteristic. Thus, we further analyzed the correlation between the S score and immune features of patients. Our results indicated that the S score was negatively correlated with the infiltration level of plasma cells, CD8 T cells, activated CD4 memory T cells, gamma delta T cells, and M1 macrophages, as well as ImmuneScore and ESTIMATE Score. Meanwhile, the S score was positively correlated with the infiltration level of memory B cells, naive CD4 T cells, M0 macrophages, M2 macrophages, and eosinophils (**Figures 6A–L**). The detailed information of the correlation between the S score component genes and immune features is shown in **Figure 6M**. These results implied that high S score predicts an immune-suppressive feature of HCC.

### The Potential of the S Score in Predicting Response to Immunotherapy of Patients

**Figure 7:**
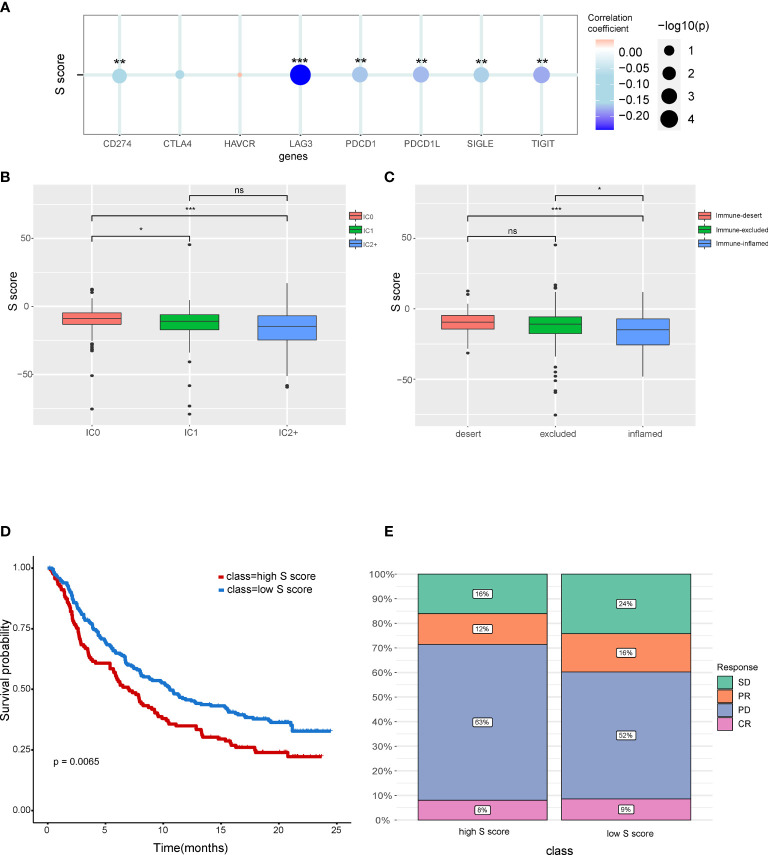
The potential of the S score in predicting response to immunotherapy in cancer. **(A)** The correlation between the S score and immune checkpoints. **(B)** Comparison of S score in different ICs (IC) groups. **(C)** Comparison of S score in groups with different immune microenvironment characteristics. Samples have been classified into three immune microenvironment-related subtypes: immune-deserted, immune-inflamed, and immune-excluded. **(D)** Comparison of prognosis of patients in high– and low–S score groups. **(E)** Distribution of responsive status in high– and low–S score groups. SD, stable disease; PD, progressive disease; PR, partial response; CR, complete response. *P < 0.05, **P < 0.01, and ***P < 0.001. NS, Not Significant.

Our result indicated that the S score can well predict the immune features in HCC. Correlation analysis indicated that the S score is negatively correlated with the expression level of immune check point related genes, which suggested that high S score may predict the low response rate to immunotherapy (**Figure 7A**). To validate our hypothesis, immunotherapy cohort imv210 was enrolled in the analysis. We found that the IC0 group has the highest S score and that the immune inflamed group has the lowest S score, which implied the high–S score group has low response rate to immunotherapy (**Figures 7B, C**). Kaplan-Meier curve also demonstrated that patients of the high–S score group had a significantly worse outcome than the low–S score group (**Figure 7D**). Patients in the high–S score group also had a higher rate of PD (**Figure 7E**). Thus, the high S score can predict the low responsiveness to immunotherapy in cancer patients. Patients with low S score would be more sensitive to immunotherapy.

## Discussion

HCC is the commonest malignant liver tumor with poor clinical outcomes (38). The heterogeneity of HCC necessitates personalized management. Senescent cells exist in tumor tissues. Senescence is an important hallmark of HCC (39). Senescence is the essential regulator of tumor immune microenvironment (40). The senescence of HCC cells would activate various types of ICs, including T cells, NK cells, and macrophages. Whereas, the activation of innate immune system promotes the clearance of HCC cells (41, 42). The different senescence status is one of the dominant reasons of the heterogeneity of HCC. Therefore, developing a senescence-related prognosis model for HCC is urgently needed.

On the basis of the characteristics of senescence, the HCC samples were classified into senescence active and senescence inactive subtypes. The immune characteristics of the two subtypes were evaluated. ESTIMATE calculations indicate that the senescence active subgroup shows lower immune activity. To further illustrate the differences of immune characteristics in the two subgroups, we calculated their specific immune characteristics using CIBERSHOT and the results provided us new insight into the association between senescence and tumor microenvironment.

The cooperation and interaction of the cancer-associated ICs determine the immune status of cancer. During cancer progression, CD8^+^ T cells suffer from dysfunction and depletion due to the immunosuppressive signals in the tumor microenvironment. In the present study, a significantly higher infiltration level of Treg cells was observed in senescence active HCC subtype (**Figure 1D**). Treg cells are one of the major promotors of the CD8^+^ T cell depletion (43, 44). Under the circumstances, although there is no significant difference in the level of CD8^+^ T cell infiltration between the two subtypes, Treg may contribute to the depletion of CD8^+^ T cells, making it an immunosuppressive subtype.

The differentially expressed genes between the two HCC subtypes were then identified. A total of 2,902 mRNAs, 19 miRNAs, and 308 lncRNAs were identified (**Figures 2A–C**). Enrichment analysis revealed the functions of differentially expressed genes. The top three enriched terms are related to metabolic and differentiation. Then, we constructed a regulatory network using differentially expressed genes between the two subgroups (**Figure 3**). Some of the hub nodes in the network play an important role in HCC. For example, the expression of miR-339 is decreased in HCC tissues and cells, whereas the enhanced expression of miR-339 can inhibit the invasion ability of HCC cells. Moreover, low miR-339 expression was correlated with poor prognosis of patients with HCC (45). The expression of miR-429 was also downregulated in HCC tissues and cells. The overexpression of miR-429 inhibited the migration and invasion of HCC cells (46). Through the ceRNA mechanism, lncRNAs and mRNAs can competitively bind specific miRNAs. The lncRNAs, miRNAs, and mRNAs in the network could interact with each other. On the basis of the network, we construed a prognosis model consisting of 26 genes for HCC by machine learning approaches. The constructed model has high accuracy in predicting prognosis of HCC in the training and four validation datasets (**Figures 4**, **5**; **Supplementary Figure 1**).

Immune-based therapies have revolutionized the treatment of HCC (47). Immunotherapy is effective for some HCC cases. However, there are still a proportion of patients that are not responding to immunotherapy (48). The stratification biomarker to predict response of patients is an unmet need. Here, we further explored the potential of the S score in predicting responsiveness to immunotherapy. The results showed that the S Score was correlated with infiltration level of ICs and already defined immune-related scores including Immune Score and ESTIMATE Score. All genes in the S score were correlated with ICs and/or immune scores (**Figure 6**).

Characteristics of immune microenvironment have clinical application potential, which could predict the response to immunotherapy. The S score was also shown to be correlated with immune checkpoints including CD274, LAG3, PDCD1L, SIGLE, and TIFIT. The high S score group has a lower response rate to immunotherapy. We also found that immune dessert cancer subtype has the highest S score. The immune status of patients can predict responses to immunotherapy. The immune-inflamed phenotype was correlated with higher responses to immune check point inhibitors (49). Consistent with these findings, our results also showed that the prognosis of the low–S score group was better than the high–S score group. Moreover, the proportion of patients in progressive disease was higher in the high–S score group compared with that in the low–S score group.

Our study still has some limitations. First, this study is based on the predicted miRNA regulatory networks. Further studies are needed to validate the functions and mechanism of key genes. Second, clinic trail is necessary for the transformation of research into clinical practice. Follow-up and high-throughput data from clinical samples are also needed for further study. Last, the present study focuses on the changes at the transcriptome level. The integration of multi-omics data will provide a comprehensively characterization of the senescence-related mechanism in HCC. We will keep working on these points in the further.

In conclusion, our study comprehensively analyzed the potential regulatory mechanism of senescence in HCC and constructed a ceRNA regulatory network. We also analyzed the correlation between senescence and the alteration of tumor immune microenvironment in HCC. Moreover, on the basis of the network, we constructed a prognostic model to predict patient prognosis, immune characteristics, and immunotherapy response. The constructed prognostic model has great clinical application potential, which will promote the precision medicine for HCC.

## Data Availability Statement

The data that support the findings of this study are available from the corresponding author upon request.

## Author Contributions

YJ, BL, and HW contributed to conception and design of the study. YJ and KL wrote or contributed to the writing of the manuscript. XS, WF, and XZ collected the data. KL, JX, and YG did the bioinformatic analysis. All authors contributed to the article and approved the submitted version. All authors contributed to the article and approved the submitted version.

## Funding

This work was supported by the National Natural Science Foundation of China (Grant Nos. 81803524 and 81803012), the Heilongjiang Postdoctoral Science Foundation (Grant Nos. LBH-Z18168 and LBH-Q21134), and College of Pharmacy, Harbin Medical University Youth Talents Start-up Funding (Grant No. 2019-QD-02).

## Conflict of Interest

The authors declare that the research was conducted in the absence of any commercial or financial relationships that could be construed as a potential conflict of interest.

## Publisher’s Note

All claims expressed in this article are solely those of the authors and do not necessarily represent those of their affiliated organizations, or those of the publisher, the editors and the reviewers. Any product that may be evaluated in this article, or claim that may be made by its manufacturer, is not guaranteed or endorsed by the publisher.

## Supplementary Material

Supplementary Table 1 Gene signatures of senescence.

Supplementary Table 2 S score component genes and their coefficients.

Supplementary Figure 1 S score distribution for patients in the four external validation cohorts. **(A)** S score distribution for patients in the GSE10143. **(B)** S score distribution for patients in the GSE14520. **(C)** S score distribution for patients in the GSE76427. **(D)** S score distribution for patients in the LIRI-JP.
