# Supplementary figures and images for: Integrated Analysis Revealing the Senescence-Mediated Immune Heterogeneity of HCC and Construction of a Prognostic Model Based on Senescence-Related Non-Coding RNA Network
Source: Front Oncol. 2022 Jun 30;12:912537. doi: 10.3389/fonc.2022.912537 (PMC9279728; doi:10.3389/fonc.2022.912537)

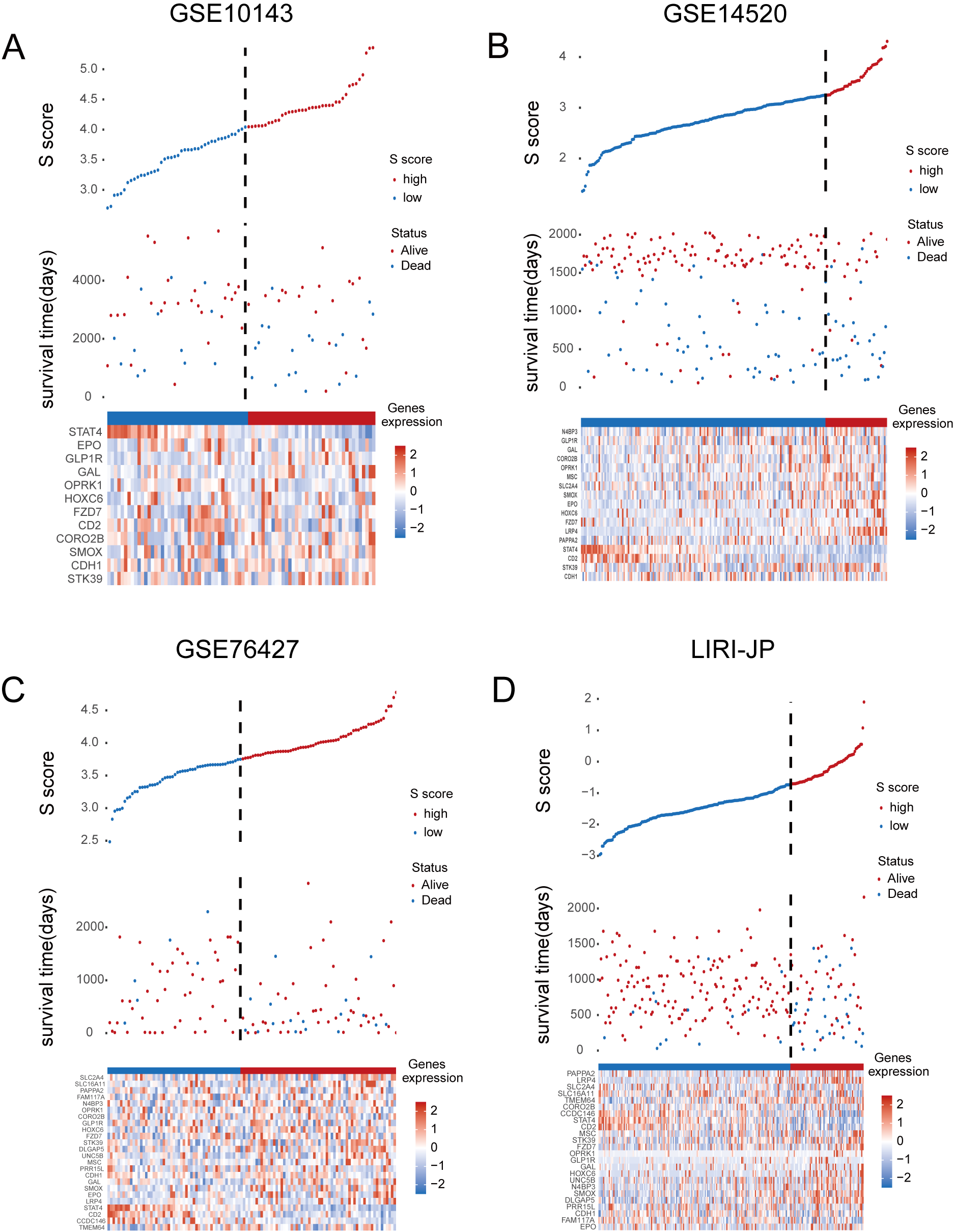

Supplement: Supplementary Figure 1 — S score distribution for patients in the four external validation cohorts. (A) S score distribution for patients in the GSE10143. (B) S score distribution for patients in the GSE14520. (C) S score distribution for patients in the GSE76427. (D) S score distribution for patients in the LIRI-JP. [file Image_1.tif]
